# Diagnostic accuracy of ChatGPT and teledentistry compared with dentist examination in detecting dental caries: preliminary diagnostic accuracy study

**DOI:** 10.1186/s12903-026-09638-z

**Published:** 2026-08-22

**Authors:** Inas Karawia, Aya Mahmoud, Rewan Ali, Marie Kamal, Aya Gaber, Mohamed Ashraf Hall

**Affiliations:** 1 Dental Public Health and Preventive Dentistry, Faculty of Dentistry, Pharos University in Alexandria, Alexandria, Egypt; 2 Faculty of Dentistry, Pharos University, Alexandria, Egypt; 3 Alexandria Dental Research Center, Ministry of Health and Population, Alexandria, Egypt

**Keywords:** Dental caries, Diagnostic accuracy, Teledentistry, Artificial intelligence, ChatGPT

## Abstract

**Introduction:**

Dental caries is a common oral health problem globally, necessitating accurate and accessible diagnostic approaches. This study aimed to evaluate and compare the diagnostic accuracy of ChatGPT (AI-based assessment) and teledentistry with the clinical dental examination as the clinical reference standard for detecting dental caries.

**Methodology:**

A diagnostic accuracy study was conducted on a sample of 40 tooth images, determined through power analysis. Standardized smartphone images were obtained and assessed independently using two approaches: ChatGPT-based analysis and teledentistry evaluation by dental professionals. The findings from both methods were compared to clinical examination results. Diagnostic performance was evaluated using sensitivity, specificity, positive predictive value (PPV), negative predictive value (NPV), accuracy, and area under the curve (AUC). Agreement with the clinical reference standard was assessed using Cohen’s kappa coefficient, while the McNemar test was used to assess systematic differences between paired classifications.

**Results:**

Teledentistry showed numerically higher diagnostic performance than ChatGPT across all evaluated parameters. It achieved higher sensitivity (89.5% vs. 78.9%), specificity (85.7% vs. 66.7%), PPV (85% vs. 68.2%), NPV (90% vs. 77.8%), and overall accuracy (87.5% vs. 72.5%). Additionally, teledentistry showed a higher AUC (0.876) compared to ChatGPT (0.728), suggesting better discriminative ability. Both methods showed no statistically significant differences from the clinical reference standard; however, ChatGPT exhibited greater inconsistency.

**Conclusion:**

Teledentistry demonstrated higher diagnostic accuracy and agreement with the clinical reference standard than ChatGPT in this preliminary study and may serve as a reliable diagnostic adjunct, particularly in remote settings. However, further refinement and validation of AI-based tools such as ChatGPT are necessary to improve diagnostic performance, with future research focusing on optimizing image quality and expanding datasets to enhance AI reliability.

**Supplementary Information:**

The online version contains supplementary material available at 10.1186/s12903-026-09638-z.

## Introduction

Dental caries of permanent teeth remains a major global and national public-health challenge, contributing significantly to disease burden and reduced quality of life [1, 2]. Despite advances in oral health care, disparities persist, particularly in low- and middle-income countries such as Egypt, where caries prevalence and untreated lesions remain high [3, 4]. Data from the Global Burden of Disease framework and national surveys highlight the need for equity-driven prevention and improved oral-health systems [1, 3, 5].

Visual examination is considered the gold standard for caries detection. Nevertheless, its application in population-based screening programs and in high-risk individuals residing in remote or underserved areas can be challenging due to travel difficulties and financial burdens. Consequently, identifying cost-effective and easily accessible diagnostic approaches that can rapidly detect caries with accuracy similar to visual examination has become an important priority [5].

Electronic communication, imaging, and information technologies, including audio, video, and store-and-forward systems, support the delivery of dental care, diagnosis, consultation, treatment, education, and the exchange of dental information [6]. Teledentistry has emerged as a promising approach for the remote assessment of oral conditions through the transmission of dental photographs and other clinical information, offering a practical alternative to conventional visual examinations, particularly for individuals residing in rural or underserved communities [7]. The two main modalities of teledentistry are synchronous communication, which involves real-time interactions through live video, and asynchronous communication, which relies on the transmission of recorded information for later evaluation [8]. By promoting communication and collaboration among oral health professionals, teledentistry supports the delivery of oral health services beyond traditional clinical settings [9, 10]. It can facilitate screening, diagnosis, treatment planning, follow-up, and patient monitoring, while also improving communication between patients and members of the dental care team [11–13]. In addition, teledentistry has the potential to reduce unnecessary travel, shorten waiting times, minimize productivity losses, and improve equity in access to dental care, thereby lowering the overall burden on healthcare systems [14, 15].

Artificial Intelligence (AI) technologies have gained increasing attention in the field of dental caries detection and diagnosis. These technologies are trained using large collections of dental images, allowing them to recognize patterns and features associated with carious lesions and subsequently assess new images with a high degree of accuracy. Predictive modeling, which relies on data-driven and statistical approaches to estimate future outcomes, has also benefited substantially from advances in AI. The integration of machine learning algorithms has improved the performance and accuracy of predictive models across various healthcare applications. A recent systematic review reported that deep learning models utilizing diverse architectural designs have considerable potential to support dentists in the identification and diagnosis of caries lesions [16]. Overall, the diagnostic performance of AI-assisted systems ranges from moderate to high, depending on the clinical condition and image quality being evaluated [17–19].

Investigations have explored the role of AI and teledentistry in dental diagnosis, particularly their potential in detecting oral diseases such as dental caries, periodontal bone loss, and periapical lesions using digital radiographs as well as intraoral and extraoral photographs [17]. The diagnostic performance of different assessment methods is often determined by their validity, which is commonly measured through sensitivity and specificity. Sensitivity describes the proportion of diseased individuals who are accurately detected by the test, while specificity represents the proportion of disease-free individuals who are correctly identified as not having the condition [20].

Recent evidence suggests a growing public exposure to and use of artificial intelligence (AI) tools, particularly ChatGPT, in Egypt. While a considerable proportion of healthcare professionals recognize the potential of such technologies, concerns remain regarding their reliability in clinical decision-making [21]. In this context, the Egyptian Ministry of Health and Population has issued warnings against reliance on AI-based diagnostic tools, reflecting increasing public engagement with these systems for seeking medical advice [22]. Additionally, the expansion of telehealth services in Egypt highlights a broader shift toward remote and technology-assisted healthcare delivery, which may also extend to AI-based tools such as ChatGPT for preliminary clinical interpretation. However, existing literature has extensively evaluated teledentistry [23] and artificial intelligence applications in dental diagnostic interpretation [24] separately, but no studies to the best of our knowledge, have directly compared the diagnostic accuracy of ChatGPT and teledentistry in dental caries detection. Accordingly, the present study aims to evaluate and compare the diagnostic accuracy of ChatGPT, a general-purpose AI model, and clinician-led teledentistry for detecting dental caries, with clinical dental examination as the clinical reference standard.

## Materials & methods

### Study design and ethical considerations

This study was designed as a preliminary comparative prospective diagnostic accuracy study to evaluate and compare two remote diagnostic approaches for dental caries detection: AI-based assessment using ChatGPT and teledentistry-based assessment performed by a qualified dentist, with conventional clinical dental examination as the reference standard. The study protocol was reviewed and approved by the institutional ethics committee in Pharos University (UREAC- 02-3-448). Written informed consent was taken from patients to use their intraoral photos for this study’s purposes. All intraoral photographs were made anonymous before the image-based evaluation. No personal details were given to ChatGPT or sent through the WhatsApp app. Only the intraoral photographs and the clinical information needed for diagnosis were shared. All steps were taken following the rules for research with human participants. The privacy of each patient and the security of the data were carefully protected during the study. All procedures were conducted in accordance with established ethical standards for research involving human participants. Patient confidentiality and data privacy were strictly maintained throughout the study.

### Sample size calculation and study setting

The required sample size was calculated a priori using G*Power software (version 3.1.9.4). The calculation was based on a two-tailed independent samples t-test comparing two groups. An effect size (Cohen’s d) of 1.02 [25, 26] was assumed, with a significance level (α) of 0.05 and a statistical power (1 − β) of 0.80. The analysis indicated that a minimum sample size of 34 tooth images was required. A total of 40 tooth images were collected from 32 patients attending the diagnostic clinic at Pharos University. Patients were selected to represent a spectrum of clinical presentations encountered in routine dental practice. More than one image could be obtained from the same patient when clinically indicated.

### Participant recruitment

Patients were consecutively recruited from those attending the diagnostic dental clinic at Pharos University during February 2025. Patients with permanent teeth who provided written informed consent were eligible for inclusion. Patients with primary teeth or those who declined to provide consent were excluded from the study. The image dataset included both sound and carious permanent molars and represented both occlusal and smooth tooth surfaces. All acquired images met the required quality for diagnostic assessment and were included in the analysis.

### Data collection procedures

#### Clinical examination (reference standard)

All patients underwent a comprehensive clinical dental examination performed by a qualified dentist. The tooth surfaces were air-dried and the examination was conducted using a mouth mirror and a WHO probe under standard dental unit lighting conditions. The presence or absence of dental caries was determined based on the WHO standard clinical diagnostic criteria [27]. The reference standard consisted of visual clinical examination only; no radiographic or other adjunctive diagnostic methods were used. These findings were considered the clinical reference against which other diagnostic methods were compared.

#### Image acquisition

Intraoral photographs were obtained for each case using the same smartphone camera (iPhone 16–48 MP) under standardized conditions to ensure consistency in image quality. Standardization included appropriate lighting, angulation, and focus to optimize visualization of tooth surfaces. The clinical examination and image acquisition were performed during the same visit. No clinical intervention occurred between the reference standard assessment and image acquisition.

### Image-Based Assessment

The same collected images were independently evaluated using two diagnostic approaches:

1. ChatGPT as an AI system: (OpenAI GPT-5.3-mini), used in its free-text interaction mode through the web interface.
2. A teledentistry platform: through WhatsApp messages.

Each method assessed the images and provided a binary diagnostic outcome according to the WHO diagnostic criteria [27], indicating the presence or absence of dental caries.

#### Diagnostic procedure

For each image, diagnostic outputs from ChatGPT and the teledentistry platform were recorded and compared with the corresponding clinical examination findings. The chief complaint and relevant dental and medical history were provided for all cases to ensure a realistic clinical context.

The image-based assessments using ChatGPT and teledentistry were performed between 15 and 22 March 2025.

For all cases, ChatGPT was given the standardized prompt (“Based on the intraoral photograph and the provided clinical information, determine whether dental caries is present or absent according to the WHO diagnostic criteria. Please answer with one of the following: ‘Caries present’ or ‘No caries”) with the intraoral image and the clinical information. Each image was looked at once in a ChatGPT session. The same standardized prompt and clinical information were used each time. This was done to make sure that previous cases did not affect the case.

#### Blinding

Assessments performed by ChatGPT and the teledentistry platform were conducted independently. The teledentistry assessment was carried out by a qualified dentist who was not involved in the clinical examination. Both image-based diagnostic methods were performed without access to the clinical examination findings. The clinical examiner remained blinded to the results of both image-based evaluations.

### Outcome measures

#### The primary outcome measures included

- Diagnostic accuracy
- Sensitivity, Specificity
- Positive and Negative Predictive Values
- Agreement and significance with the clinical reference standard

These measures were used to evaluate the performance of each diagnostic modality in detecting dental caries.

### Statistical analysis

Data were analyzed using SPSS version 25. Sensitivity, specificity, positive predictive value (PPV), negative predictive value (NPV), accuracy, area under the receiver operating characteristic curve (AUC), and 95% confidence intervals were calculated to evaluate the diagnostic performance of ChatGPT and teledentistry relative to the clinical diagnosis. Receiver operating characteristic (ROC) curves and the corresponding AUC values were generated by comparing the binary diagnostic outcomes (caries present/no caries) of each diagnostic method with the clinical reference standard. Agreement was assessed using Cohen’s kappa coefficient [28], while McNemar’s test was used for paired comparisons. Statistical significance was set at *p* < 0.05. Analyses were performed for the overall sample and stratified by tooth surface type (occlusal and smooth surfaces). No missing data occurred and all index-test and reference-standard assessments yielded definitive binary outcomes (caries present or no caries). Therefore, no indeterminate results occurred, and no special handling of indeterminate results was required.

**Fig. 1:**
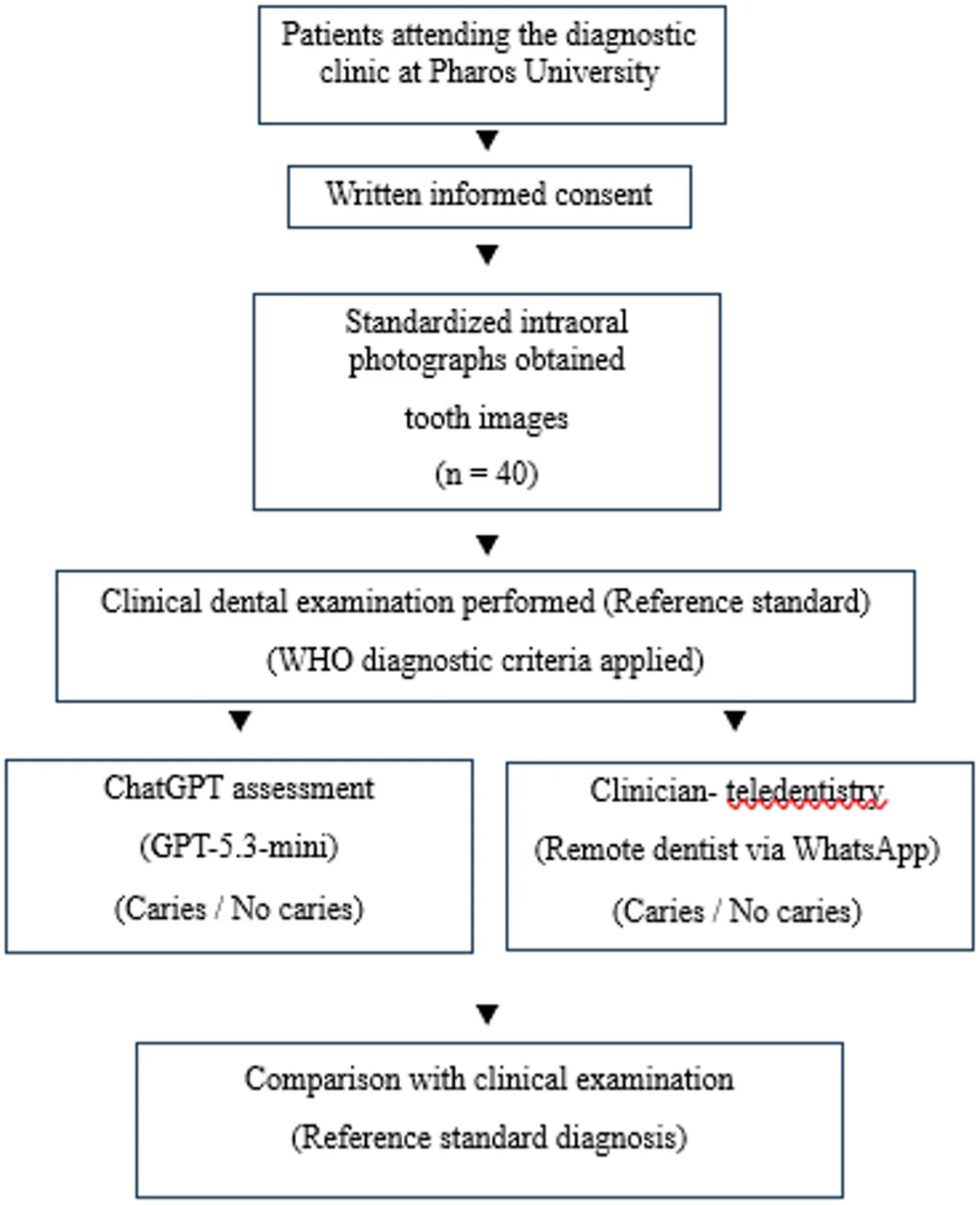
STARD flow diagram of participant selection, image acquisition, image-based assessment, and comparison with the clinical reference standard

The overall study workflow is illustrated in the STARD flow diagram (Fig. 1), and the study is reported in accordance with the STARD 2015 guidelines [29].

## Results

A total of 32 patients (mean age: 34.9 ± 9.69 years) contributed 40 tooth images for analysis. The study population comprised 13 males (40.6%) and 19 females (59.4). The image dataset consisted of permanent molars, including 19 (47.5%) carious teeth and 21 (52.5%) sound teeth. Of the 40 images, 25 (62.5%) represented occlusal surfaces and 15 (37.5%) represented smooth surfaces.

Teledentistry consistently demonstrated higher diagnostic performance than ChatGPT across all surface types. For overall performance, teledentistry showed higher sensitivity (89.5%), specificity (85.7%), accuracy (87.5%), and AUC (0.876) than ChatGPT (78.9%, 66.7%, 72.5%, and 0.728, respectively). This pattern was also observed in subgroup analyses of occlusal and smooth surfaces, where teledentistry achieved higher or comparable values, particularly on smooth surfaces, with an AUC of 0.938 and a sensitivity of 100%.

The cross-tabulations of the ChatGPT and teledentistry results against the clinical reference standard are presented in Supplementary Tables S1 and S2.

**Fig. 2:**
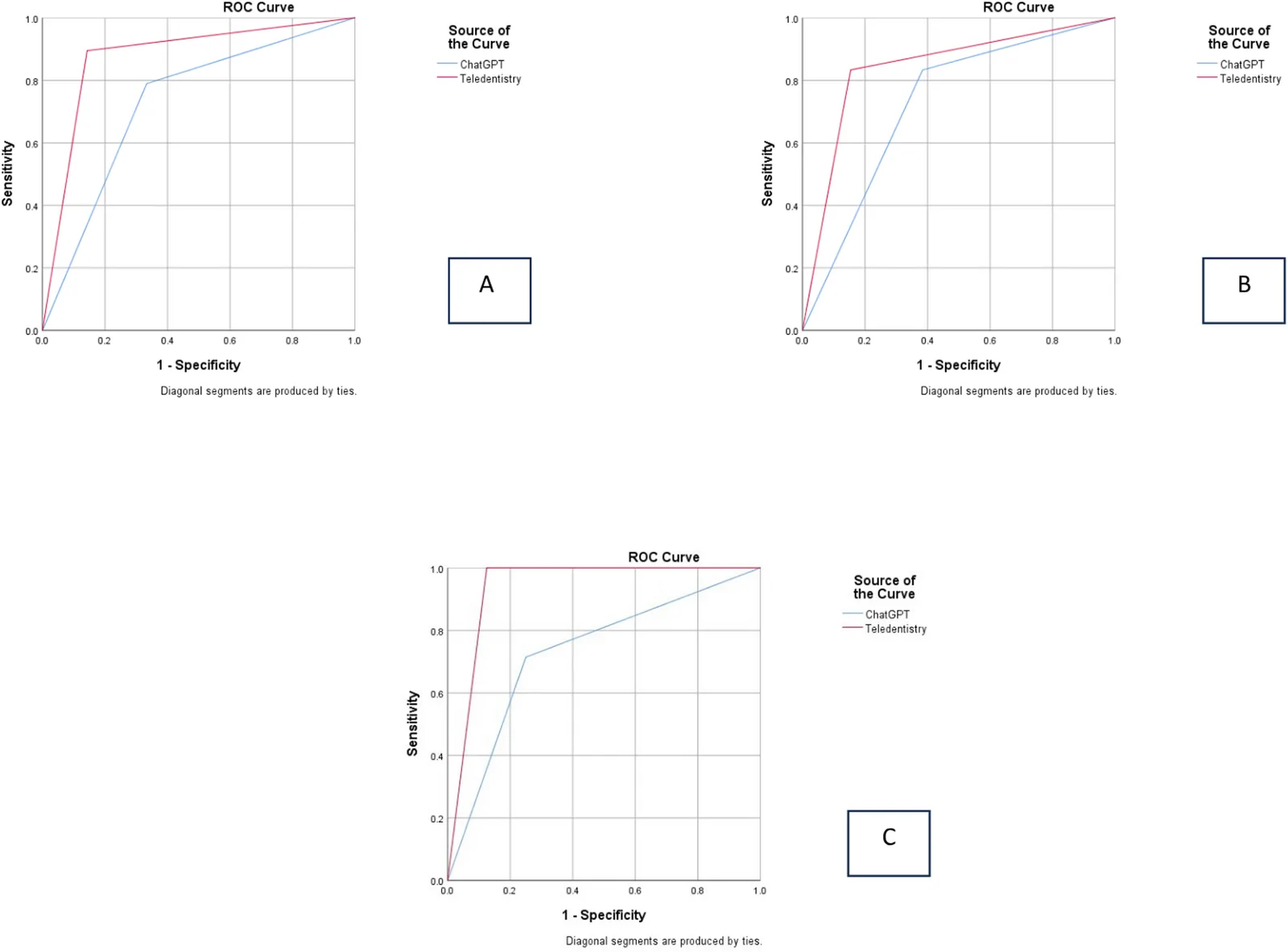
ROC curves comparing ChatGPT and Teledentistry across all surfaces (**A**), occlusal surfaces (**B**), and smooth surfaces (**C**) against the clinical standard diagnosis

Agreement with the clinical reference standard was higher for teledentistry (kappa = 0.75) than for ChatGPT (kappa = 0.453), indicating substantial agreement for teledentistry and moderate agreement for ChatGPT. McNemar tests were not statistically significant across all comparisons, indicating no significant disagreement between each diagnostic method and the reference standard (Table 1; Fig. 2).

**Table 1:** Diagnostic performance of ChatGPT and Teledentistry across different tooth surfaces compared to the clinical reference standard

Metric	All Surfaces ChatGPT	95% CI	All Surfaces Teledentistry	95% CI	Occlusal ChatGPT	95% CI	Occlusal Teledentistry	95% CI	Smooth ChatGPT	95% CI	Smooth Teledentistry	95% CI
Sensitivity	78.9	56.7–91.5	89.5	68.6–97.1	83.3	55.2–95.3	83.3	55.2–95.3	71.4	35.9–91.8	100.0	64.6–100
Specificity	66.7	45.4–82.8	85.7	65.4–95.0	61.5	35.5–82.3	84.6	57.8–95.7	75.0	40.9–92.9	87.5	52.9–97.8
PPV	68.2	47.3–83.6	85.0	64.0–94.8	66.7	41.7–84.8	83.3	55.2–95.3	71.4	35.9–91.8	87.5	52.9–97.8
NPV	77.8	54.8–91.0	90.0	69.9–97.2	80.0	49.0–94.3	84.6	57.8–95.7	75.0	40.9–92.9	100.0	64.6–100
Accuracy	72.5	57.0–84.1	87.5	73.9–94.5	72.0	52.4–85.7	84.0	65.3–93.6	73.3	48.0–89.1	93.3	70.2–98.8
AUC	0.728	0.57–0.88	0.876	0.75–1.00	0.724	0.52–0.88	0.840	0.64–0.95	0.732	0.46–0.90	0.938	0.70–0.99
Kappa	0.453	0.18–0.73	0.750	0.55–0.95	0.444	0.10–0.78	0.679	0.39–0.97	0.464	0.02–0.91	0.867	0.62–1.00
McNemar (p)	0.549	—	1.000	—	0.453	—	1.000	—	1.000	—	1.000	—

## Discussion

The present diagnostic accuracy study evaluated the performance of teledentistry and the ChatGPT artificial intelligence model as two remote diagnostic approaches of caries lesions, using direct clinical examination by a dentist as the reference standard. The findings demonstrated that teledentistry achieved diagnostic accuracy comparable to conventional clinical examination and showed higher diagnostic performance measures than the AI-based assessment generated by ChatGPT. These results highlight the growing clinical value of teledentistry as a reliable adjunctive diagnostic modality for caries lesions, particularly in remote areas.

The present findings demonstrated that teledentistry consistently showed higher values than ChatGPT across all evaluated diagnostic indicators, including sensitivity, specificity, accuracy, AUC, and agreement with the clinical reference standard. These differences should be interpreted as descriptive, as no direct statistical comparison between the two diagnostic approaches was performed. The strong diagnostic agreement between teledentistry and in-person dental examinations may be attributed to the capacity of experienced clinicians to evaluate clinical photographs while considering factors such as lesion appearance, location, patient history, and overall clinical presentation as both modalities ultimately rely on clinician-based interpretation. Although the examining dentist and the remote evaluator may differ in physical interaction with the patient, the diagnostic reasoning process remains grounded in professional clinical judgment. A systematic review published in 2024 reported similar diagnostic accuracy between teledentistry and conventional visual examination across numerous studies, providing substantial evidence for the effectiveness of teledentistry, particularly in settings with limited resources or restricted access to oral healthcare services [30]. Moreover, ongoing technological developments, including improved smartphone camera quality and the integration of artificial intelligence (AI), are expected to further enhance the diagnostic performance of teledentistry systems in the future [17]. These findings also strengthen the evidence supporting remote assessment of oral lesions and highlight the growing potential of teledentistry to expand access to specialist care, especially for underserved and geographically remote communities.

In contrast, the ChatGPT model demonstrated lower observed diagnostic accuracy than both teledentistry and direct clinical examination. Several factors may account for this outcome. First, large language models and multimodal AI systems are not specifically developed or validated solely for the diagnosis of dental caries. Consequently, their ability to differentiate between clinically similar carious lesions may be limited, particularly in cases that require interpretation of surface texture changes. Despite that, in the present study, all images were captured using the same smartphone cameras under standardized conditions to ensure consistency in image quality, including controlled lighting, appropriate angulation, and optimal focus for clear visualization of tooth surfaces, AI-based diagnoses can be affected by factors such as image quality, incomplete clinical information, and variations in lesion presentation. Second, both ChatGPT and teledentistry rely on image-based assessment and therefore share certain inherent limitations, including the inability to perform tactile examination or directly assess lesion texture. However, unlike teledentistry, ChatGPT does not benefit from clinician expertise, professional judgment, or the ability to interpret visual findings within a broader clinical context. These differences may have contributed to the lower diagnostic performance of ChatGPT observed in the present study. The moderate diagnostic performance achieved by ChatGPT in the present study is consistent with observations reported by Alhaidry et al. [31], which discussed both the opportunities and limitations of large language models in dental practice. The review described ChatGPT as a promising educational and decision-support tool capable of assisting with patient communication, clinical documentation, and preliminary guidance. However, it also highlighted significant concerns regarding diagnostic reliability, factual inaccuracies, hallucinations, and lack of true clinical reasoning. These limitations may explain the lower specificity (66.7%) and lower diagnostic accuracy (72.5%) observed in the current study. The relatively lower specificity suggests that ChatGPT tended to overdiagnose carious lesions, increasing false-positive findings and potentially leading to unnecessary referrals or overtreatment.

In contrast to the results of the current study, previous literature has described artificial intelligence systems as promising diagnostic adjuncts [17]. The current study demonstrated lower observed diagnostic accuracy of the ChatGPT AI model than teledentistry, suggesting that general-purpose AI systems may not yet achieve the reliability of professional remote clinical evaluation in oral lesion diagnosis. The apparent discrepancy between the present findings and previous optimistic reports regarding AI in oral healthcare may be explained by methodological and technological differences. Many earlier studies evaluated specialized AI applications, such as Overjet, DentalMonitoring, and Diagnocat, which were trained using dedicated dental datasets and designed specifically for oral diagnostic tasks [17], whereas the present study assessed a general-purpose multimodal AI model in a real-world clinical context. Additionally, factors such as variability in image quality, lesion complexity, limited clinical context, and prompt-dependent responses may have contributed to the lower diagnostic performance observed with ChatGPT.

Despite the lower accuracy of ChatGPT than teledentistry observed in the present study, the AI system still demonstrated potential utility as a supportive screening or triage tool. Artificial intelligence may help assist non-specialists in preliminary assessment and facilitate early referral pathways. In resource-limited settings where access to dental specialists is restricted, AI-assisted screening combined with teledentistry could contribute to earlier detection of oral pathologies. However, based on the present findings, general-purpose AI systems should not currently be considered a replacement for professional clinical judgment.

The findings of this study have important clinical implications. Teledentistry may represent a practical and cost-effective strategy for expanding oral healthcare accessibility while maintaining acceptable diagnostic reliability. This is particularly relevant in rural communities, areas with limited specialist availability, and situations where face-to-face consultation is difficult. In addition, teledentistry may improve interdisciplinary communication and reduce delays in diagnosis and referral [13].

Nevertheless, several limitations should be acknowledged. The study may have been influenced by the spectrum of included carious lesions. Furthermore, the diagnostic performance of ChatGPT may vary depending on prompt design, image quality, and future model updates. Another limitation is that limited textural information, and the inherently complex nature of caries diagnosis may have reduced the diagnostic performance of for all lesions, which could affect the certainty of the diagnosis. Future research should focus on the integration of AI-assisted systems with clinician-led teledentistry workflows. Training AI models on dedicated oral pathology datasets may improve diagnostic performance and clinical applicability. Longitudinal studies assessing cost-effectiveness, and patient outcomes would also provide valuable insight into the role of digital diagnostic technologies in oral healthcare.

## Conclusion

Within the limitations of this preliminary study, teledentistry demonstrated diagnostic accuracy comparable to direct dentist examination and higher diagnostic performance than the ChatGPT model in the assessment of dental lesions. These findings support the use of teledentistry as a reliable diagnostic adjunct, while highlighting the current limitations of general-purpose AI-only approaches. Artificial intelligence may serve as a supportive tool in caries lesion screening; however, clinical interpretation by trained dental professionals remains essential for accurate diagnosis and patient management.

## Supplementary Information

Supplementary Material 1.

## Data Availability

The datasets used and/or analyzed during the current study are available from the corresponding author on reasonable request.
