# Supplementary material for: Diagnostic accuracy of ChatGPT and teledentistry compared with dentist examination in detecting dental caries: preliminary diagnostic accuracy study
Source: BMC Oral Health. 2026 Aug 22;26:1533. doi: 10.1186/s12903-026-09638-z (PMC13499293; doi:10.1186/s12903-026-09638-z)
Supplement: Supplementary file 1 [file 12903_2026_9638_MOESM1_ESM.docx]

| **Clinical reference standard** | **ChatGPT**  **(+)** | **ChatGPT**  **(−)** | **Total** |
| --- | --- | --- | --- |
| **Caries (+)** | 15 | 4 | 19 |
| **Caries (−)** | 7 | 14 | 21 |
| **Total** | 22 | 18 | 40 |

**Table S1. Cross-tabulation of ChatGPT versus the clinical reference standard**

**Table S2. Cross-tabulation of Teledentistry versus the clinical reference standard**

| **Clinical reference standard** | **Teledentistry**  **(+)** | **Teledentistry**  **(−)** | **Total** |
| --- | --- | --- | --- |
| **Caries (+)** | 17 | 2 | 19 |
| **Caries (−)** | 3 | 18 | 21 |
| **Total** | 20 | 20 | 40 |
